# Modeling the distribution of the endangered Jemez Mountains salamander (*Plethodon neomexicanus*) in relation to geology, topography, and climate

**DOI:** 10.1002/ece3.9161

**Published:** 2022-08-23

**Authors:** Andrew W. Bartlow, J. Tomasz Giermakowski, Charles W. Painter, Paul Neville, Emily S. Schultz‐Fellenz, Brandon M. Crawford, Anita F. Lavadie‐Bulnes, Brent E. Thompson, Charles D. Hathcock

**Affiliations:** ^1^ Biosecurity & Public Health Group Los Alamos National Laboratory Los Alamos New Mexico USA; ^2^ Museum of Southwestern Biology 1 University of New Mexico Albuquerque New Mexico USA; ^3^ Earth Data Analysis Center 1 University of New Mexico Albuquerque New Mexico USA; ^4^ Endangered Species Program, New Mexico Department of Game and Fish Santa Fe New Mexico USA; ^5^ Earth and Environmental Sciences Division Los Alamos National Laboratory Los Alamos New Mexico USA; ^6^ Environmental Stewardship Group Los Alamos National Laboratory Los Alamos New Mexico USA

**Keywords:** Bandelier Tuff, endangered species, habitat suitability, Maxent, species distribution model, Valles caldera

## Abstract

The Jemez Mountains salamander (*Plethodon neomexicanus*; hereafter JMS) is an endangered salamander restricted to the Jemez Mountains in north‐central New Mexico, United States. This strictly terrestrial and lungless species requires moist surface conditions for activities such as mating and foraging. Threats to its current habitat include fire suppression and ensuing severe fires, changes in forest composition, habitat fragmentation, and climate change. Forest composition changes resulting from reduced fire frequency and increased tree density suggest that its current aboveground habitat does not mirror its historically successful habitat regime. However, because of its limited habitat area and underground behavior, we hypothesized that geology and topography might play a significant role in the current distribution of the salamander. We modeled the distribution of the JMS using a machine learning algorithm to assess how geology, topography, and climate variables influence its distribution. The best habitat suitability model indicates that geology type and maximum winter temperature (November to March) were most important in predicting the distribution of the salamander (23.5% and 50.3% permutation importance, respectively). Minimum winter temperature was also an important variable (21.4%), suggesting this also plays a role in salamander habitat. Our habitat suitability map reveals low uncertainty in model predictions, and we found slight discrepancies between the designated critical habitat and the most suitable areas for the JMS. Because geological features are important to its distribution, we recommend that geological and topographical data are considered, both during survey design and in the description of localities of JMS records once detected.

## Graphical abstract

The Jemez Mountain salamander is an endangered salamander in northern New Mexico. We used Maxent to determine habitat suitability based on geological, topographical, and environmental variables. We found that geological features and climate variables are important to its distribution, and we recommend that these variables be considered in future conservation efforts.

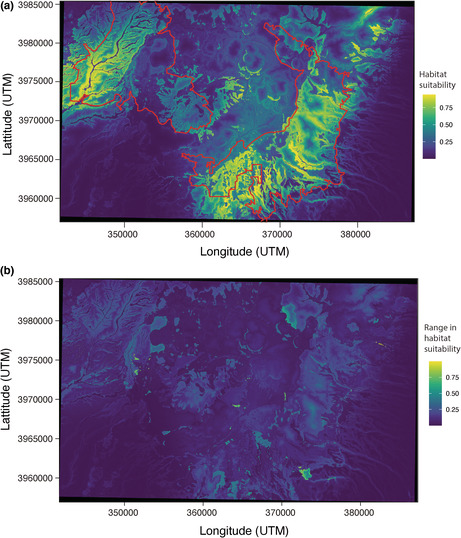

## 1 INTRODUCTION

Assessing the distributional extent of taxa is essential for species that are endemic, rare, and have limited dispersal capabilities, all of which increase their risk of extinction (Chunco et al., 2013; Smith & Green, 2005). This is because an accurate evaluation of habitat suitability informs management and conservation decisions by helping to identify potential areas to survey and protect, saving time and resources (Ancillotto et al., 2020; Crawford et al., 2020). Species Distribution Models (SDMs) help to evaluate habitat suitability and are key to estimating risk to species by understanding potential vulnerabilities and what landscape and climate variables contribute most to their distribution (Wang et al., 2020). These tools can also be useful for predicting species' responses to future climate and environmental change (Beest et al., 2021; Pang et al., 2021), which is especially important for climatically constrained species, such as those that live in mountainous areas (Ali et al., 2021; Rahbek et al., 2019; Xenarios et al., 2019).

The Jemez Mountains salamander (*Plethodon neomexicanus*, hereafter referred to as JMS) is endemic to the Jemez Mountains in north‐central New Mexico and found primarily around the flanks and rim of the Valles‐Toledo caldera complex in mixed‐conifer forests (Degenhardt et al., 1996). It is a relatively rare and strictly terrestrial salamander and is the only *Plethodon* species in New Mexico. Like other members of the family Plethodontidae, the JMS is highly fossorial and lungless, requiring moist conditions for surface activity, such as foraging or mating. It was listed as endangered under the Endangered Species Act in 2013 because of large and severe wildfires due to extensive drought (U.S. Fish and Wildlife Service, 2013).

The current federally delineated critical habitat of the JMS consists mostly of mixed‐conifer forests. Principal threats to JMS habitat include historical fire exclusion and suppression, severe wildland fires, forest composition and structure conversions, postfire rehabilitation, forest and fire management, roads, trails, habitat fragmentation, recreation, and disease (U.S. Fish and Wildlife Service, 2013). Jemez Mountains salamanders spend long periods of time underground, presumably in voids caused by the local geology, plant roots, or other processes; however, little is known about their underground habitat requirements. They move very little and have an estimated home range of 8 m^2^ (Ramotnik, 1988). Because of their limited movements and underground behavior, we hypothesize that the geology and topography might play a significant role in their current distribution. Additionally, a recent study assessed the habitat suitability of members of the genus *Plethodon* in the Pacific Northwest and found that the distributions of these species depend strongly on precipitation (Nottingham & Pelletier, 2021) but the study's climate focus means it did not evaluate whether geologic and/or soil conditions contributed to species distribution.

Maxent is a type of machine learning SDM that uses a maximum entropy probability distribution to contrast occurrence data (i.e., presence records) with environmental data, such as climate, soil type, and geology, and estimates a probability distribution that has the maximum entropy (i.e., that is most spread out, or uniform) given certain constraints. The constraints are that the expected values of each feature, such as a climate variable, must equal the average value at known occurrence points, or most common value for categorical variables (Phillips et al., 2006). Maxent is one of the best algorithms to calculate the suitability of landscape for a species when presence/absence data are not available (Elith et al., 2006; Elith & Leathwick, 2009). This is particularly the case for animals such as the JMS, because its absence is difficult to confirm due to its cryptic habits and low detectability. Models built with presence‐only data do not incorporate information on the frequency of occurrence, and therefore cannot accurately predict probability of presence (Guisan & Thuiller, 2005; MacKenzie et al., 2002). However, such models can be used to estimate an index of the suitability of landscape for a species, including the relative importance of different variables (Elith et al., 2006).

Here, we provided an assessment of to what extent the geology, topography, and certain climate variables in the Jemez Mountains and around the Pajarito fault system influence the distribution of the JMS. We first compiled all available records of the occurrence of the salamander. We constructed Maxent models of potential habitat suitability at fine scales (5 m), by including features of geology, topography (i.e., elevation, slope, and topographical complexity from a LiDAR‐derived digital elevation model [DEM]), and seasonal summaries of climate. We evaluated the relative importance of these climatic, geologic, and LiDAR‐derived features in models of habitat suitability and determined which were meaningful and important parameters for identifying JMS habitat.

## 2 MATERIALS AND METHODS

### 2.1 Study area

**FIGURE 1:**
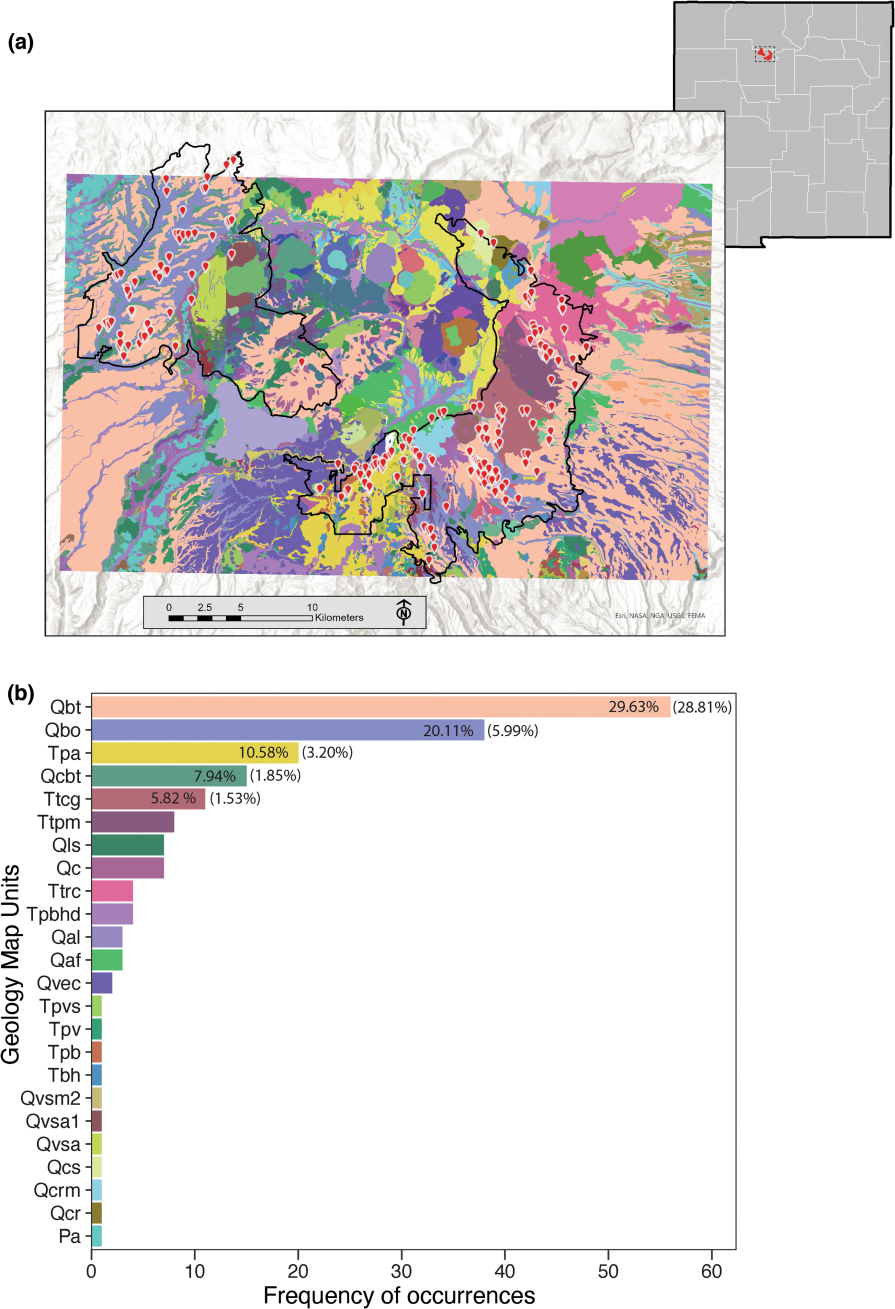
(a) Geologic map and area of analysis for modeling habitat suitability of the Jemez Mountains salamander in north‐central New Mexico. The red points are localities where salamanders were detected in surveys or collected as specimens. These represent the thinned presence points (*n* = 189) that were used in the modeling process. This area includes the U.S. Fish and Wildlife Service federally designated critical habitat extent (outlined in black). (b) Number of salamander occurrences according to geology map units. The geology map unit colors in (b) match those in (a). Numbers in bars represent the percentage of salamander occurrences. Numbers in parentheses indicate the percentage of that geological map unit in our study area. Only those map units with greater than 10 salamander occurrences are presented. Descriptions of the map units in B can be found in the Appendix A.

The Jemez Mountains, situated in north‐central New Mexico, includes the federally designated critical habitat of the JMS (Figure 1) and encompasses the Valles caldera, a resurgent volcanic caldera (Smith & Bailey, 1968). This volcanic system has been active since ca. 23 Ma (Gardner & Goff, 1984; WoldeGabriel et al., 2003), with two large ash‐flow tuff‐producing, caldera‐forming eruptions at 1.6 and 1.25 Ma (age from Phillips et al., 2007), respectively. The two large ash‐flow tuff‐producing eruptions created the Bandelier Tuff, which blankets all of the surrounding flanks of Valles caldera. Subsequent smaller rhyolitic eruptions continued until 50–72 ka (Gardner et al., 1986; Wolff et al., 2011; Zimmerer et al., 2016). The Jemez Mountains and Valles caldera are separated from the Pajarito Plateau in the east by the Pajarito fault system, a dominantly down‐to‐the‐east complex normal fault that is potentially seismogenic and presently defines the active western margin of the Española Basin of the Rio Grande rift (Gardner et al., 1999; Lewis et al., 2002, 2009). Elevation within the critical habitat ranges between 2200 and 3100 m (U.S. Fish and Wildlife Service, 2013). The eastern portion of the JMS habitat was recently subject to multiple large fires, some with high intensity and high severity burns and as large as 63,400 ha.

The climate within the JMS critical habitat is continental and semi‐arid, with precipitation dominated by convective storms between July and September. Salamanders rely on this summer precipitation and resulting moisture for above‐surface activity (Degenhardt et al., 1996). Winter precipitation is highly variable year to year due to Pacific Ocean teleconnections (Sheppard et al., 2002). Total annual precipitation within the critical habitat averages 643 mm (1981–2010; PRISM Climate Group, 2019). Annual average minimum temperature is −2.4 C, whereas the average maximum temperature is 12.3 C (1981–2010; PRISM Climate Group, 2019).

### 2.2 Compilation of JMS presence records

We contacted several colleagues at different agencies and universities for data on surveys and any records of Jemez Mountains salamanders. In particular, we relied on field notes of the late Charles W. Painter (CWP; New Mexico Department of Game and Fish herpetologist) and other data deposited with the Museum of Southwestern Biology (MSB) at the University of New Mexico in association with JMS specimens. We queried, processed, and used information from Los Alamos National Laboratory (LANL; Hathcock et al., 2017), New Mexico Natural Heritage (NMNH), and the Global Biodiversity Information Facility (GBIF) data aggregator. We were able to compile 655 records from GBIF representing 49 distinct localities and reconciled these data with 690 records from NMNH, of which 650 were those documenting presence of the salamander. All presence records were georeferenced. The records of JMS span from the years 1949 to 2017 and at each locality the number of salamanders documented ranges from 0 to 110 (mean = 4.86, median = 2) (110 = type locality, from where the species was described).

### 2.3 Geological, topographical, and climate data

We used ten variables in our distribution models [geologic: (1) unit classification based on 1:24,000 scale geologic maps produced by New Mexico Bureau of Geology and Mineral Resources, New Mexico Institute of Mining and Technology (Gardner et al., 2006; Goff et al., 2002, 2012; Goff, Gardner, et al., 2006; Goff, Reneau, et al., 2006; Kelley et al., 2004; Kempter et al., 2002; Timmer et al., 2006), (2) distance to the boundary of mapped geologic contacts (Goff et al., 2011) within the Valles caldera region; topographic: (3) high‐resolution elevation (10 m), (4) slope, (5) topographic characterization (i.e., curvature; change in slope, first derivative) from a LiDAR‐derived digital elevation model (DEM); climatic: (6) total precipitation in summer, (7) total precipitation in winter, (8) maximum temperature in winter, (9) minimum temperature in winter, (10) minimum temperature in summer].

**FIGURE 2:**
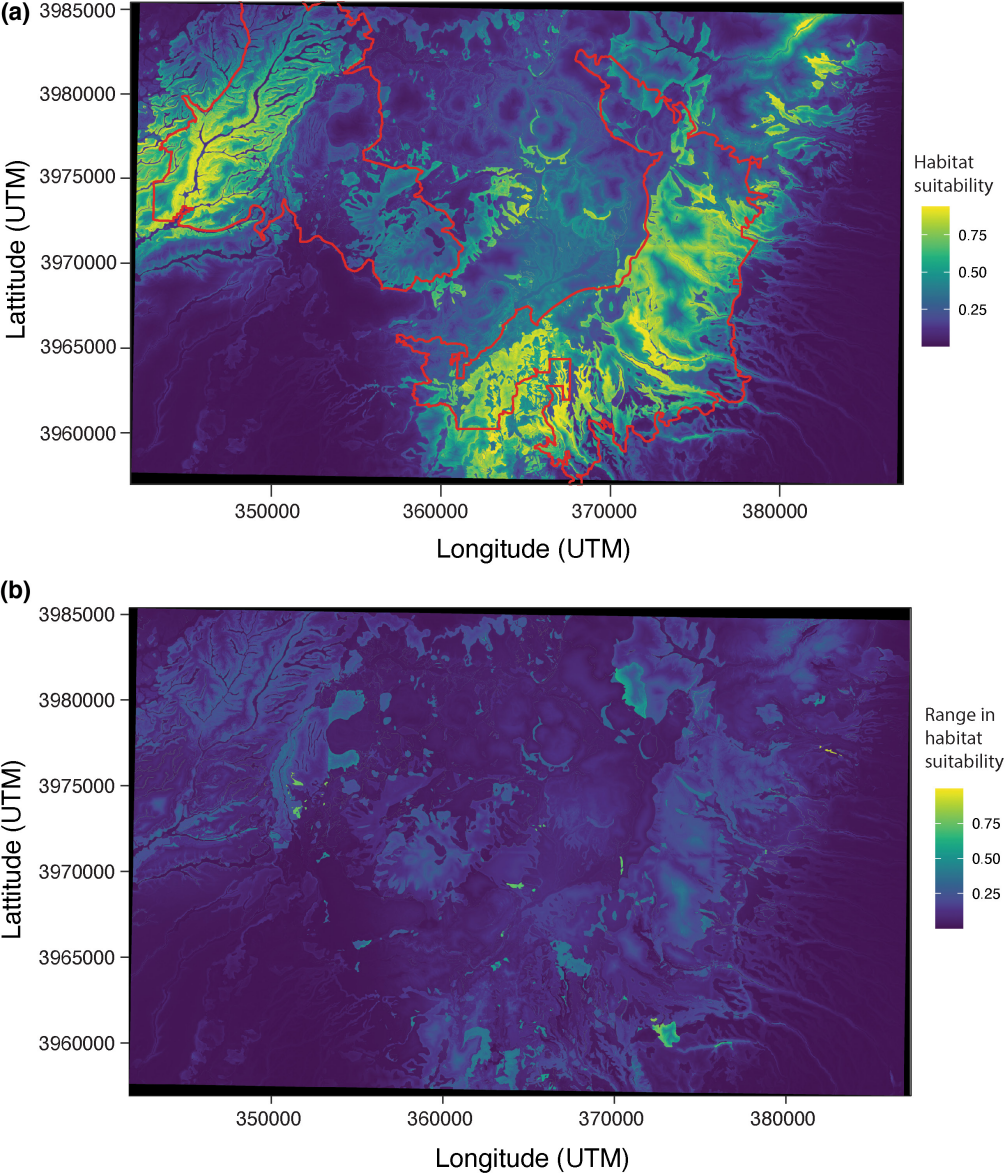
(a) Habitat suitability map for JMS from the Maxent models. Colors indicate the mean habitat suitability from the 10 bootstrapped Maxent models using the parameters of the top model after model selection. The northern part of the range is not included in the map due to lack of fine‐scale geological data available for modeling. Yellow colors indicate areas with high habitat suitability, while darker blue colors indicate areas with lower habitat suitability. The red outline is the federally designated critical habitat designated by the U.S. Fish and Wildlife Service. (b) Map of the study area depicting the uncertainty in habitat suitability for the Jemez Mountains Salamander. Colors indicate the range in maximum and minimum values in habitat suitability from the 10 bootstrapped Maxent models using the parameters of the top model after model selection.

Geologic unit classification was a categorical variable (Figure 2; Appendix A). Geologic unit classification can be generalized into a few principal groups: the Quaternary‐aged rhyolitic Bandelier Tuff, which is further divided into the older Otowi Member and the younger Tshirege Member (Qbo and Qbt, respectively); various Tertiary‐aged andesitic to dacitic volcanic units (Tpa, Ttcg, Ttpm, etc.); and other surficial Quaternary geomorphic deposits including landslides (Qls), alluvial fans (Qaf, Qal), colluvium (Qc, Qcbt), and others (Appendix A).

The climate variables were PRISM‐derived southwest‐specific climate variables based on climate normals (1981–2010; PRISM Climate Group, 2019). They consisted of total precipitation in the summer months (July to September; locally known as the monsoon season), total precipitation in the winter months (November to March), maximum temperature in the winter months (November to March), minimum temperature in the winter months (November to March), and minimum temperature in the summer months (April to October; 1981–2010; PRISM Climate Group, 2019). Climate data was at 800 m resolution, and when needed (50% of our variables were downscaled), we downscaled the data to match our intended 5 m fine scale for our analysis. To downscale the coarse scale PRISM data to 5 m, we followed the methodology of Lee et al. (2014). We believe that climate data, because of its continuous nature, is least likely to introduce bias at such scales.

### 2.4 Modeling JMS distribution

We modeled the current extent of suitable habitats for JMS using Maxent implemented in the ENMeval package (Kass et al., 2021; version 2.0.0) within the R statistical framework (R Core Development Team, 2021). We intentionally set the area of analysis to include the federally designated critical habitat for the JMS (colored region of Figure 1a). We used this entire extent during the modeling and habitat suitability mapping process in order to find suitable habitats within, and outside, the federally designated critical habitat. The very northern part (<10 km^2^) of federally designated critical habitat is not included in the analyses due to a lack of fine‐scale geological data needed for modeling. Because of the gridded nature of analyses and models, we used all point coordinates and associated errors of JMS records as pixels representing presence of JMS (e.g., coordinates with a 10 m error would mean that 9 pixels are treated as if JMS was present there). In cases of multiple records, we only used one set of coordinates to minimize sampling bias, which is especially important for correlative modeling (Phillips et al., 2009). In addition, we spatially thinned the occurrences to 100 m using the thin function in the spThin package (Aiello‐Lammens et al., 2015; version 0.2.0). Spatial thinning resulted in 189 presence points for modeling.

We used the maxent.jar algorithm (maxent.jar v3.4.1 from the dismo package Hijmans et al., 2020; version 1.3.3) for the models using the ENMevaluate function in the ENMeval package (Kass et al., 2021; version 2.0.0). The importance of different variables was evaluated for inclusion in the final model using a variety of different feature classes and regularization multipliers. To develop the final distribution model, we considered linear (L), quadratic (Q), and hinge (H) feature classes (in machine learning language, features are transformations of variables into functions). We included hinge features because they produce model projections similar to those based on Generalized Linear Models (GLMs) or Generalized Additive Models (GAMs), but allow different fits to different parts of the response (as opposed to GLMs or GAMs, which only describe one response; Elith et al., 2010; Phillips & Dudík, 2008). We did not select product (P) features (interactions of variables) because of the complexity in the ecological interpretation of interacting variables, and we did not select threshold (T) features because those tend to be redundant with hinge features (Elith et al., 2010).

We tested 4 types/combinations of feature classes: L, H, LQ, and LQH. We also tested four regularization multipliers; 0.5, 1, 2, and 5, which resulted in 16 total models. The higher the regularization multiplier, the higher the penalty for models with higher numbers of variables; thus, larger values encourage models with fewer covariates, lowering overfitting. All environmental variables were continuous variables, except for geological classification, which was set as a categorical variable.

We initiated Maxent to randomly sample 10,000 background points within our entire extent and trained the models using k‐fold cross‐validation using the jackknife partitioning method. Although choice of background data can have important effects on predictions (VanDerWal et al., 2009), a large number of locations (10,000) from a broad range of conditions in the Jemez Mountains was used to ensure good representation of all possible environments, which is important when models are to be projected into different conditions (Elith et al., 2010).

We selected the best model using a combination of the area under the curve of the receiver‐operating characteristic (AUC), omission rates, and AICc values. We use a custom stepwise process to select models to avoid overfitting (Gorris et al., 2021). First, we selected only half the models with the lowest omission rates using the 10% omission rate of the training localities metric (or.10p.avg). Omission rates greater than the expected 10% usually means that the model overfits the data (Muscarella et al., 2014). From these models, we selected models that had the lowest difference between the training and testing AUC (auc.diff.avg) (Gorris et al., 2021). These models were chosen based on being lower than the median value of auc.diff.avg across the remaining models, which was 0.102. After the above steps, we then chose the model with the lowest AICc value as the top model. We assessed the included predictor variables in the top model using the built‐in permutation importance and percent contribution. Together, these metrics allow for the identification of important predictors in the model (Cobos et al., 2019; Gorris et al., 2021).

After selecting the best model, we assessed the uncertainty in our predictions using the feature class and regularization multiplier of the best model. For each of 10 replicates, we bootstrapped our presence data (*n* = 189) using 80% of our presence data. We used the difference between the minimum and maximum habitat suitability (i.e., the range) among the 10 bootstrapped replicates to show areas of lower and higher uncertainty in our models (Gorris et al., 2021; Romero‐Alvarez et al., 2020). For all models, we used 10,000 background points and the jackknife method of cross‐validation.

We created a habitat suitability map for the entire extent using the mean values of the 10 bootstrapped models. We used the predict function to create a habitat suitability map (occurrence intensities) for each of the bootstrapped models. We used the complementary log–log (cloglog) transformation to give us probabilities of presence. We used these transformed model outputs because they fall between 0 and 1. However, the term “probability of presence” is subject to a few assumptions about the sampling scheme (Phillips et al., 2017). Therefore, we describe the outputs as “relative habitat suitability” with 0 being low habitat suitability, 0.5 being medium suitability, and 1.0 being high suitability (Gorris et al., 2021). The permutation importance and percent variable contribution are reported as the means from all 10 bootstrapped models.

## 3 RESULTS

### 3.1 JMS presence in relation to coarse‐scale geology and LiDAR data

After compiling data on presence of salamanders throughout the Jemez Mountains, we first examined the relationships between occurrence and coarse geological unit information based on the 1:24,000 scale geologic maps produced by New Mexico Bureau of Geology and Mineral Resources (Figure 1a) as well as topographic data and analyses derived from fine‐scale (1 m) LiDAR imagery. Each discrete geologic unit correlates with colors and labels used in Figure 1a,b; detailed geologic unit descriptions and abbreviations for regions where species observations have been documented are listed in the Appendix A. We intentionally set the area of analysis to include the federally proposed critical habitat for the salamander (Figure 1).

Nearly 50% of localities where salamanders were detected are within the 1.6 Ma Otowi and 1.25 Ma Tshirege Members of the Bandelier Tuff (Qbo and Qbt, respectively) (Figure 1), with the next closest unit, the Paliza Canyon Formation andesite flows having around 11% of occurrence localities (Tpa; Figure 1). However, within the designated critical habitat and surrounding areas, 34.8% of the area consists of Bandelier Tuff members Qbo and Qbt, and 3.2% consists of Tpa. Thus, salamanders have been recorded disproportionately in areas associated with those geologic units in relation to their availability, hinting at a pattern.

Furthermore, analyses of LiDAR data indicate that localities where salamanders have been detected are concentrated in topographically complex areas. Based on data derived from LiDAR, the average slope of sites where JMS have been recorded is 42.06% (*SD* = 23.09, range = 0–89), whereas the average change in slope for those sites is 35.13% (*SD* = 19.11, range = 0–77.6) (LiDAR curvature, first spatial derivative). Thus, univariate examination of salamander records indicates that those records are concentrated in certain geological areas (associated with Bandelier Tuff) that are somewhat steep and where the slope changes abruptly. However, these coarse comparisons only suggest patterns with geology and topography and do not consider other variables, such as those concerning climate.

### 3.2 Modeling JMS distribution

We used Maxent to find the most important geological, topographical, and climate variables important for the distribution of the JMS. The top model for JMS habitat suitability had a linear and quadratic (LQ) feature class, a regularization multiplier of 2, and 35 model parameters (Table 1). The selection of this model was based on a custom set of thresholds in order to not overfit the data.

**TABLE 1:** Summary of the four top Maxent models that passed the omission rate and difference between training and test AUC thresholds (see Section 2). Included here are types of feature classes, regularization multipliers, AUC for training data, AICc values, the deviation from the best model (ΔAICc), and number of model parameters. The top model has a feature class of LQ, a regularization multiplier of 2, and 35 parameters.

Feature class	Regularization multiplier	Train AUC	AUC mean difference	Mean OR 10%	SD OR 10%	AICc	ΔAICc	Number of parameters
LQ	2	0.894	0.099	0.121	4.483	6340.62	32.17	35
H	2	0.910	0.090	0.148	4.871	6396.93	88.48	62
LQH	2	0.909	0.089	0.148	4.871	6404.48	96.01	64
LQH	5	0.875	0.102	0.116	4.397	6429.82	121.38	30

Abbreviations: AICc. Akaike information criterion; AUC, area under the curve; OR, omission rate; SD, standard deviation.

This top model was used for the 10 bootstrapped replicates in order to make a habitat suitability map and determine permutation importance and percent contribution. The habitat suitability map shows areas in the Valles caldera and surrounding Jemez Mountains that have varying degrees of habitat suitability (Figure 2a). The northern part of federally designated critical habitat is not included in the map due to lack of fine‐scale geological data available for modeling. Most of the suitable habitat was within the currently designated critical habitat. Areas of high suitable habitat were also identified outside of the designated critical habitat, most notably in the northeastern portion of our study area and places between the two critical habitat areas (red outline; Figure 2a). Only a few regions reveal higher model uncertainty (Figure 2b). Higher uncertainty suggests interpretation within these areas should be approached with more caution. However, this model is relatively robust; most areas within our study area have very low ranges in habitat suitability, meaning that all iterations of the model produced very similar results.

The variable with the highest percent contribution was geological classification (45.6%) followed by maximum temperature during the winter months (26.3%; Table 2). The variable with the highest percent permutation importance is maximum temperature during the winter months (50.3%) followed by geological classification (23.5%; Table 2), suggesting that geology and winter climate is an important component of their distribution. Minimum temperature in winter months was third for both percent contribution and permutation importance. Few variables are not important for the distribution of the JMS (Table 2). Elevation, curvature, slope, distance to the boundary of mapped geologic contacts, minimum temperature in summer, and the two precipitation variables had low mean values for both percent contribution and permutation importance (all <8%).

**TABLE 2:** Mean percent contribution and mean permutation importance for all variables of the 10 bootstrapped Maxent distribution models. These mean values are based on bootstrapping the top Maxent model shown in Table 1.

Variable	Percent contribution	Percent permutation importance
Elevation	0.09	0.93
Curvature	0	0
Distance to boundary of mapped geologic contacts	3.52	1.15
Geological unit classification	45.58	23.50
Slope	7.20	0.96
Total precipitation in summer	0.79	0.15
Total precipitation in winter	1.94	1.63
Maximum temperature in winter	26.33	50.30
Minimum temperature in summer	3.87	0
Minimum temperature in winter	10.69	21.39

## 4 DISCUSSION

Most Plethodontid salamanders have limited ranges and many species need protection due to habitat vulnerability (Milanovich et al., 2010). The Jemez Mountains salamander is endemic to New Mexico and more specifically to the flanks of the Valles caldera in mixed‐conifer forests (Degenhardt et al., 1996). Further threats to this federally listed endangered species include declining or changing forest cover, changing fire regimes resulting in less frequent but more severe fire, increases in temperatures of soil, and associated evaporation, and changes in precipitation patterns (U.S. Fish and Wildlife Service, 2013). These stressors underlie the importance of understanding and to what extent the geology, topography, and certain climate variables in the Jemez Mountains and around the Pajarito fault system influence the distribution of the JMS.

Several studies test geological variables regarding habitat suitability in plants and animals, and geological variables are known to rank relatively high in Maxent models (reviewed in Bradie & Leung, 2017). For underground species, it is especially important to consider these variables. However, climate and other variables, such as distance to water, soil type, and anthropogenic factors, may also play important roles in species distributions in primarily underground species (Bradie & Leung, 2017).

To our knowledge, this is the first study that examines the suitability of the landscape throughout the Jemez Mountains for the namesake salamander at a relatively fine scale (5 m) and considers geological, topographical, and climate data as variables in determining habitat suitability. Our analyses indicate that geology of the Jemez Mountains and climate variables influence the distribution of the endangered JMS. Geological classification contributed over 45% to the top model, followed by maximum temperature in winter months (26%). Both of these variables were the top two in terms of permutation importance as well with winter temperature being the most important variable (50.3%). In both percent contribution and permutation importance, minimum temperature in winter months ranked third. Together, these three variables have a combined 82.6% contribution and 95.2% permutation importance.

Geological classification and winter temperature could both impact where JMS is able to live during winter months. For instance, the minimum and maximum temperature in the winter months likely determines how far down in the ground freezing occurs; and thus, where the salamander is able to persist throughout the winter. Areas that are frozen would be inaccessible. The geological classification may play a role in this as well, determining which areas remain unfrozen and are suitable for salamanders during the winter months.

Contrary to studies of other *Plethodon* species, precipitation did not influence the habitat suitability of the JMS (Camp et al., 2014; Nottingham & Pelletier, 2021). Subsurface voids around faults may be moist enough, given that this species does not require standing water for development. It is known that species in this genus are known to occupy distinct niches and that their distributions and their biotic and abiotic habitat requirements are difficult to predict (Pelletier & Carstens, 2016). Since this is the only *Plethodon* species in New Mexico, and has been generally understudied, perhaps this species has relatively unique landscape and climate requirements compared to other species.

Upon initial inspection, we found that sites where JMS have been recorded are relatively steep (the average slope is 42% or 19 degrees), and in topographically complex areas (the average change in slope [curvature] is 35%). Examination of salamander records indicates that they are concentrated in certain geological areas (associated with Bandelier Tuff) that are somewhat steep and where the slope changes abruptly. However, slope and curvature were not important variables in the top Maxent model, highlighting the advantage of species distribution modeling in determining habitat suitability. Additionally, this suggests that for the JMS, geology is sufficient to capture their habitat requirements and that slope and curvature can be ignored.

In some portions of the study area, topographic complexity and geology can be correlated, suggesting a potential importance of such areas for the JMS. For example, a geologically related element, but one that is not incorporated as a model input, is the presence or absence of geologic structures such as faults, folds, and fracture zones. These features can create abrupt topographic changes and subsurface voids, which can be inhabited by salamanders. Within the eastern sectors of the JMS critical habitat, the Pajarito fault system is present within Qbt and younger Quaternary geomorphic units and creates abrupt topographic changes and subsurface voids that could account for the very high average slope observations in locations where JMS have been recorded. This suggests that the specific geology surrounding faults should be given priority for surveys and conservation efforts. This is also corroborated by the maps produced from results of the Maxent modeling; areas around the Pajarito fault system appear to have high habitat suitability.

There were several geologic map units in which salamander occurrences were greatest. Qbt (Bandelier Tuff, Tshirege Member) had the most salamander occurrences (29.6%). This is consistent with this geology type being the most frequent in our study area (28.81%; Figure 2b). However, the geology types Qbo, Tpa, Qcbt, and Ttcg also had high salamander occurrences. For these areas, salamanders are more numerous than the frequency of these geology types in our study area (ratio of salamanders to geology type >3), suggesting that salamanders congregate in these geology types since they are disproportionately inhabited by salamanders. The occurrence of JMS in certain geology types may represent a correlation with certain soil characteristics. Geology is often a coarse surrogate for soil characteristics, which are often an important component of the habitat of certain salamander species. In addition to the geological and topographical variables considered here, future work should consider additional variables, such as more accurate soil composition, pH, and moisture retention (Nottingham & Pelletier, 2021). More fine‐scale soil characteristics may allow for greater accuracy in predictions of salamander landscape requirements. Because geological features play an important role in JMS distribution, we recommend that future surveys take geological data into account, both during study and survey design and in the description of localities of JMS records once detected.

Our study suggests that geological features may exert an important influence on the distribution of JMS. Consequently, it is important to re‐evaluate the current extent of the JMS critical habitat designated by the U.S. Fish and Wildlife Service (2013). There is a slight discrepancy between the current designated critical habitat and the most suitable areas for the JMS, which suggests that it should be slightly expanded to include areas northeast of the current designation and areas to the south. However, designation of critical habitat considers many other factors beyond a species' habitat requirements, including known occurrence data as well as economic and environmental impacts of the designation (U.S. Fish and Wildlife Service, 2013).

Applying the latest techniques to produce multiple iterations of models, as well as current advances in evaluating and selecting models with appropriate statistics, makes our results robust. A few places had higher uncertainty (range in habitat suitability values) than others, although the bootstrap process produced very similar results for most of our study area, including the federally designated critical habitat. In order to improve our JMS distribution model and to potentially reduce uncertainty, there are two areas of further work to enhance our input parameters that we believe would improve the accuracy of predicting JMS occurrence. First, a refinement of individual home range estimates and movements, as well as determining numbers of individuals throughout patches of their occurrence would help improve model accuracy. Second, the calibration of distribution models with calculated detectability and occurrence data at fine scales would further improve modeling efforts. Our approach relies on data collected over many decades with varying degrees of error. Fine scale modeling could benefit from surveyor‐grade (<1 m or better) placement of records and areas searched for JMS. We expect that as precise survey‐grade, fixed GPS units become available, surveys for the JMS habitat will greatly benefit.

Our goal was to determine if, and to what extent, geology, topography, and certain climate variables in the Jemez Mountains influence the distribution of the JMS. We created habitat suitability maps and found relatively low uncertainty in our predictions. We found that geological classification as well as maximum and minimum winter temperatures are the most important variables in the JMS distribution. Coarse relationships between known salamander occurrences and topography suggested that topography variables (slope and curvature) should be important variables for their habitat suitability. However, these variables were determined to not be important, meaning that these variables can be ignored in the future. Based on our results, geological classification can be used instead since it is much more informative. Future work should consider geology in species distribution modeling, especially in species that live underground.

## AUTHOR CONTRIBUTIONS

**Andrew W. Bartlow:** Formal analysis (supporting); methodology (equal); writing – original draft (equal). **J. Tomasz Giermakowski:** Data curation (lead); formal analysis (lead); methodology (equal); writing – original draft (equal). **Paul Neville:** Data curation (supporting); methodology (supporting); writing – review and editing (equal). **Emily S. Schultz‐Fellenz:** Conceptualization (equal); funding acquisition (lead); project administration (lead); writing – review and editing (equal). **Brandon M. Crawford:** Project administration (supporting); writing – review and editing (equal). **Anita F. Lavadie‐Bulnes:** Writing – review and editing (equal). **Brent E. Thompson:** Investigation (equal); writing – review and editing (equal). **Charles D. Hathcock:** Conceptualization (equal); investigation (equal); writing – review and editing (equal).

## CONFLICT OF INTEREST

The authors declare no conflicts of interest.

## Data Availability

Maxent data files are hosted on Dryad (https://doi.org/10.5061/dryad.9ghx3ffkz). Due to the Jemez Mountains salamander being endangered and the sensitive nature of the data, we did not include geolocation data (presence records) of where the salamanders were found.

## APPENDIX A

Abbreviations of the map units used for Figure 1a,b only where there are documented occurrences of Jemez Mountain salamanders. Map unit names follow the conventions described by the North American Commission on Stratigraphic Nomenclature (2005). First capital letter describes the age of the unit; the second letter generally describes the unit lithology; and subsequent letters identify details of the specific geologic unit. Example: Qbt = Quaternary‐aged Bandelier Tuff, Tshirege Member. All descriptions, except one (Qc/Qcbt; Kempter et al., 2004), are aggregated from Goff et al. (2011); unit thicknesses are specific to the areas covered by those studies. References in this table can be found in Goff et al. (2011).

**Table:**

Map unit	Description
Pa	**Permian Abo Formation**—Brick‐red to dark‐red, medium‐ and thin‐bedded, arkosic, cross‐stratified, fluvial sandstone; interbedded with micaceous siltstone and mudstone. The basal portion of the Abo Formation is dominated by mudstones; channel sands become thicker and more abundant in the upper part of the formation. Thin pedogenic carbonate beds are common on and just south of Cerro Colorado; maximum thickness about 260 m on Cerro Colorado
Qaf	**Quaternary Alluvial fans (late Holocene to late Pleistocene)**—Typically fan‐shaped deposits of coarse to fine gravel and sand, silt, and clay within and at the mouths of valleys and inside north and east caldera margins; some fan deposits (Qafu) are difficult to distinguish from older alluvial fans (described below); maximum exposed thickness about 15 m
Qal	**Quaternary Alluvium (mostly Holocene)**—Deposits of sand, gravel, and silt in main valley bottoms; maximum thickness may exceed 15 m
Qbo	**Quaternary Bandelier Tuff, Otowi Member**—Poorly to densely welded rhyolitic ash‐flow tuff; originated from catastrophic eruptions that formed Toledo caldera; pumice and matrix contain abundant phenocrysts of sanidine and quartz, and sparse mafic micropheno‐crysts; sanidine may display a blue iridescence; contains abundant accidental lithic fragments; basal Guaje Pumice Bed to east described by Bailey et al. (1969) not found in map area; 40Ar/39Ar ages 1.61 ± 0.01 to 1.62 ± 0.04 Ma (Izett & Obradovich, 1994; Spell et al., 1996); magnetic polarity reverse; maximum exposed thickness about 120 m
Qbt	**Quaternary Bandelier Tuff, Tshirege Member**—Multiple flows of densely welded to nonwelded rhyolitic ash‐flow tuff erupted during formation of the Valles caldera (Smith & Bailey, 1996, Smith & Bailey, 1968); pumice and matrix contain abundant phenocrysts of sanidine and quartz, sparse microphenocrysts of clinopyroxene and orthopyroxene, and extremely rare microphenocrysts of fayalite (Warshaw & Smith, 1988; Warren et al., 2007); in more welded portions, sanidine typically chatoyant (blue iridescence); contains accidental lithic fragments of older country rock; locally has a thin (<2 m) laminated, pumice‐fall and surge deposit at base of unit (Tsankawi Pumice Bed) that contains roughly 1% of hornblende dacite pumice (Bailey et al., 1969); most recent 40Ar/39Ar age determination is 1.25 ± 0.01 Ma (Phillips et al., 2007); magnetic polarity reverse; maximum observed thickness within caldera more than 900 m. On the eastern flanks of the Jemez Mountains, Qbt has been further divided into multiple subunits (Broxton & Reneau, 1995) but are grouped as Qbt for mapping at this scale
Qc/Qcbt (Kempter et al., 2004)	**Quaternary Colluvium. Late Pleistocene to Holocene**. Poorly sorted talus, debris, and colluvium in wedge‐shaped deposits on hill slopes. Numerous hill slopes beneath mesas of Tshirege Member, Bandelier Tuff (Qbt), are covered by Qbt colluvium (obscuring the underlying bedrock), and have been mapped as Qcbt. Thickness can locally exceed 5 m
Qcr	**Quaternary Aphyric rhyolite**—Two dome and flow complexes and two small intrusive bodies of flow‐banded lava; obsidian phases are completely aphyric and probable source of artifacts (Steffen, 2005); devitrified phases contain spherulites and very sparse microphenocrysts of quartz, sanidine, and biotite; K–Ar age of dome northwest of Cerro Rubio (Ttcr) is 1.33 ± 0.02 Ma (Stix et al., 1988); maximum exposed thickness is 365 m
Qcrm	**Quaternary Rabbit Mountain rhyolite**—Large dome with thick flows and flow breccias of aphyric to sparsely porphyritic obsidian to white, devitrified lava; obsidian is a known source of artifacts (Steffen, 2005); actual vent area is probably northwest of location shown on map; vent collapsed before or during formation of Valles caldera; small exposure of associated bedded tuff (Qcrmt) is southwest of dome; 40Ar/39Ar age is 1.428 ± 0.007 Ma; maximum exposed thickness about 410 m
Qcs	**Quaternary Sierra de Toledo rhyolite**—Flow‐banded, sparsely porphyritic lava with phenocrysts of quartz, sanidine, biotite, and tiny magnetite; sanidine is typically chatoyant blue; possibly originates from two vents; 40Ar/39Ar ages of two samples range from 1.34 to 1.38 Ma (Spell et al., 1996); maximum exposed thickness is 365 m
Qls	**Quaternary Landslides (late Holocene to late Pleistocene)**—Poorly sorted debris that has moved chaotically down steep slopes; includes slumps or block slides that are partially to completely intact; thickness varies considerably depending on the size and nature of the landslide
Qvec	**Quaternary El Cajete Pyroclastic Beds**—Moderately sorted beds of pyroclastic fall and thin pyroclastic flow deposits; rhyolite pumice clasts contain sparse phenocrysts of plagioclase, quartz, and biotite with rare microphenocrysts of hornblende and clinopyroxene; unit dated at about 50–60 ka (Toyoda et al., 1995; Reneau et al., 1996) to 72 ka (Zimmerer et al., 2016); magnetic polarity normal (Geissman, 1988); maximum exposed thickness varies from 70 m in vent area to scant exposures too thin to map
Qvsa/Qvsa1	**Quaternary San Antonio Mountain Member**—Flow‐banded, massive to slightly vesicular rhyolite lavas containing phenocrysts of sanidine, plagioclase, quartz, biotite, hornblende, and clinopyroxene; consists of two main flow units based on morphology (Qvsa2 and Qvsa1) erupted from San Antonio Mountain; a third flow and peripheral vent (Qvsa3) may be present at Sulfur Point; 40Ar/39Ar age of Qvsa1 is 0.557 ± 0.004 Ma (Spell & Harrison, 1993); magnetic polarity normal; maximum exposed thickness at least 510 m
Qvsm2	**Quaternary South Mountain Member**—Flow‐banded, massive to slightly vesicular porphyritic rhyolite lavas containing abundant phenocrysts of sanidine, plagioclase, quartz, biotite, hornblende, and clinopyroxene in a pale‐gray, perlitic to white, devitrified groundmass; apparently consists of four flow units based on morphology (youngest to oldest Qvsm4 to Qvsm1); fills paleocanyon in southern moat of Valles caldera; 40Ar/39Ar age of Qvsm2 is 0.52 ± 0.01 Ma (Spell & Harrison, 1993); maximum exposed thickness is at least 450 m. Includes Cerro La Jara rhyolite (Qvlj), a small dome of flow‐banded, massive to slightly vesicular porphyritic lava; 40Ar/39Ar age is 0.53 ± 0.01 Ma (Spell & Harrison, 1993); magnetic polarity normal; maximum exposed thickness about 75 m
Tbh	**Tertiary Bearhead Rhyolite**—Dikes, plugs, and flows of aphyric to slightly porphyritic, devitrified to completely silicified rhyolite containing sparse phenocrysts of quartz, sanidine, plagioclase, biotite, opaque oxides ± hornblende; locally shows pervasive hydrothermal alteration consisting of quartz, chalcedony and/or opal, illite, Fe‐ and Mn‐oxides, pyrite, and possibly other sulfides, alunite, jarosite, and gypsum; 40Ar/39Ar ages on widely separated samples range from 4.81 to 7.83 Ma (Justet & Spell, 2001; Kempter et al., 2007) maximum observed thickness about 100 m
Tpa	**Tertiary Two‐pyroxene andesite, undivided**—Domes, flows, flow breccia, spatter deposits, and scoria of andesite from multiple sources; vents are widely scattered; individual units are slightly porphyritic to very porphyritic containing phenocrysts of plagioclase, orthopyroxene, and clinopyroxene; alteration varies from slight to intense consisting of silica, calcite, Fe‐oxides, clay ± chlorite ± zeolite ± pyrite ± epidote; 40Ar/39Ar ages in western and southern map area range from 8.2 to 9.4 Ma (Justet, 2003); maximum exposed thickness about 150 m
Tpb	**Tertiary Olivine basalt and basaltic andesite, undivided**—Flows, flow breccia, spatter deposits, and scoria of basalt and subordinate basaltic andesite from multiple vents; most units are slightly porphyritic containing phenocrysts of olivine, plagioclase ± clinopyroxene; displays variable amounts of hydrothermal alteration consisting of silica, calcite, Fe‐oxides, clay ± zeolite ± chlorite ± epidote ± pyrite; 40Ar/39Ar ages from western and southern map areas range from 8.88 to 9.45 Ma (Justet, 2003); maximum exposed thickness about 150 m
Tpbhd	**Tertiary Porphyritic biotite, hornblende dacite**—Extensive dome and flow complex filling paleocanyon south of Rabbit Mountain; contains large phenocrysts of plagioclase, plus biotite, hornblende, orthopyroxene, and clinopyroxene; contains vesiculated enclaves of plagioclase, pyroxene ± hornblende ± biotite as large as 30 cm in diameter; hydrothermally altered to clay, silica, calcite, Fe‐oxides, chlorite ± epidote; 40Ar/39Ar age is 8.66 ± 0.22 Ma; exposed thickness at least 275 m
Tpv	**Tertiary Volcaniclastic member (Pliocene? to Miocene)**—Tpv is conglomeratic sandstone and sandy conglomerate locally containing cinder deposits, pyroclastic‐ fall deposits, and lava flows too small or thin to map; unit has accumulated in small basins, topographic lows, and paleocanyons; contemporaneous with eruption of lavas of the Paliza Canyon Formation; upper part of unit may be correlative with oldest Cochiti Formation (Smith & Lavine, 1996); maximum exposed thickness about 70 m
Tpvs	**Tertiary volcaniclastic sandstone**—Tpvs is a moderately to well‐sorted volcaniclastic sandstone that is brick red to tan and contains mostly volcanic fragments, feldspar, mafic minerals, and minor quartz; present between lava flow contacts in isolated locations throughout southeastern part of the map area; mapped only where laterally extensive and at least 3 m thick
Ttcg	**Tertiary Cerro Grande dacite**—Extensive dome and flow complex of massive to sheeted, porphyritic lava containing phenocrysts of plagioclase, hypersthene, and (typically) conspicuous hornblende; the latter two phases commonly show oxidized rims that may be difficult to see in hand sample; ages on widely separated samples range from 2.88 to 3.35 Ma (Broxton et al., 2007); maximum exposed thickness is about 750 m
Ttpm	**Tertiary Pajarito Mountain dacite**—Dome and flow complex of massive to sheeted, porphyritic lava containing phenocrysts of plagioclase, hypersthene, clinopyroxene, and opaque oxides in a devitrified groundmass; 40Ar/39Ar ages on geographically separated samples range from 2.93 to 3.09 Ma (Broxton et al., 2007); maximum exposed thickness is about 365 m
Ttrc	**Tertiary Rendija Canyon rhyodacite**—Dome and flow complex of massive to sheeted, highly porphyritic lavas with phenocrysts of quartz, sanidine, plagioclase, hornblende, and biotite; 40Ar/39Ar ages on widely separated samples range from 3.50 to 5.36 Ma (Broxton et al., 2007); maximum exposed thickness approximately 500 m
